# Isolation, characterisation and detection of breath-derived extracellular vesicles

**DOI:** 10.1038/s41598-020-73243-5

**Published:** 2020-10-15

**Authors:** Garima Dobhal, Amrita Datta, Deanna Ayupova, Paul Teesdale-Spittle, Renee V. Goreham

**Affiliations:** 1 School of Mathematical and Physical Sciences, University of Newcastle, Callaghan, 2308 Australia; 2 School of Chemical and Physical Sciences, Victoria University of Wellington, Wellington, 6012 New Zealand; 3 School of Biological Sciences, Victoria University of Wellington, Wellington, 6012 New Zealand; 4 Centre for Biodiscovery, Victoria University of Wellington, Wellington, 6012 New Zealand

**Keywords:** Nanoscience and technology, Nanobiotechnology, Nanoscale materials

## Abstract

The physical characterisation, capture and detection of extracellular vesicles (EVs) and exosomes derived from breath condensate is reported. Breath-derived EVs were isolated from breath condensate and captured on a gold substrate using two complimentary methods. The characterised and isolated EVs were detected using surface plasmon resonance (SPR) and electrochemical impedance spectroscopy (EIS). EIS was done using aptamers as a targeting moiety and showed a larger change in resistance between dilute concentrations of EVs (less than 7 μg/mL).This is the first report of EVs and exosomes isolated and characterised from breath. In addition, EVs from a non-invasive and easily available source such as breath opens up further avenues in the detection of pulmonary diseases.

## Introduction

Exhaled breath has been a key biological media used to assess human health, along with urine and blood. Each person has the capacity to exhale 10,000 L of breath each day, making exhaled breath condensate (EBC) an attractive matrix that contains accessible biomarkers for the diagnosis of diseases^1–4^. It has been shown that EVs from EBC are a novel and non-invasive avenue for pulmonary disease detection^5^. To date, however, the physical characterisation of isolated EVs and exosomes from EBC has not been done despite previous evidence of the presence. EVs, in particular exosomes, are nano-sized vesicles secreted by most living cells. Previously thought to be leftover debris, literature has demonstrated that they can play a vital role in cell-to-cell transport, cell function and intercellular communication due to their contents which are specific to the cell of origin^6,7^. They have shown massive potential as biomarkers for a wide range of diseases, for example, cancer and PRION-related diseases, as they are found in most bodily fluids, including blood^4^, saliva^8,9^, breast milk^10^, and urine^11^. EVs from EBC are, therefore, a feasible target to analyse when compared to small molecules (such as volatile organic compounds), as they are a larger size of 40–100 nm (making them easier to detect, capture and characterise), highly abundant and have accessible and specific target proteins on the surface. The detection of EVs using plasmonic and electrochemical sensors has been done previously using specific cell-line derived EVs^12,13^. Kilic et al.^13^ showed the use of an electrochemical sensor to measure EVs isolated from a MCF-7 cell line. However, this is the first attempt at detecting of EVs isolated from EBC with low available concentrations. In this work, the presence was verified by isolating and physically characterising EBC-derived EVs according to the “minimal information for studies of EVs” (MISEV) requirements^14^. Sinha et al.^5^ verified the presence of an exosome-specific biomarker collected from EBC (CD63) but did not characterise the physical properties which were achieved here using photoluminescence spectroscopy, transmission electron microscopy (TEM), cryo-scanning electron microscopy (cryo-SEM), western blot and dynamic light scattering (DLS). Then, the EVs were detected from EBC using different techniques such as SPR, using fluorescent InP/ZnS quantum dots (QD) conjugated to an EV-specific antibody (AB), and EIS, using an EV-specific aptamer. The above detection protocols can be translated to disease-specific EBC-derived EVs for the purposes of disease detection and a breathalyser.

## Results and discussion

**Figure 1:**
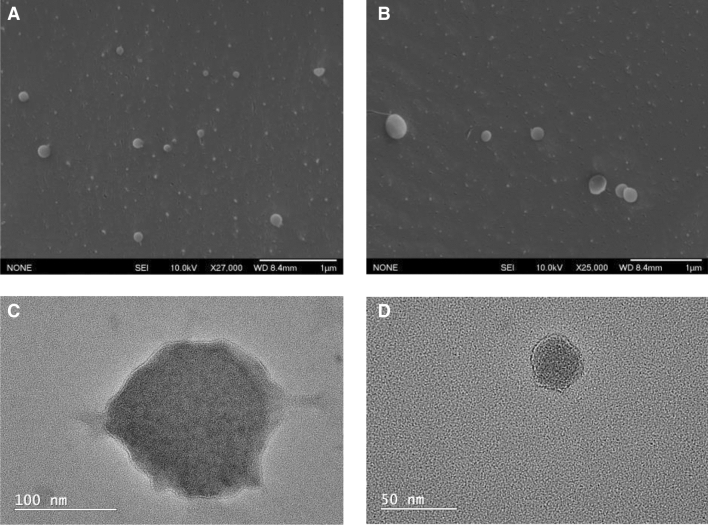
(**A**,**B**) Cryo-SEM of breath EVs. (**C**,**D**) High-resolution TEM of breath EVs.

For the isolation of EBC-derived EVs, breath condensate was first isolated using a frozen column to condense the breath. A size-exclusion column was then used to produce highly pure EVs with minimal protein aggregates. To confirm the presences of EV, characterisation was done using TEM and Cryo-SEM to investigate their morphology and size. According to both the cryo-SEM and TEM images (shown in Fig. 1), the size of the EVs was varied and observed to be anywhere between 50 to 170 nm. The morphology was observed to be spherical and the isolation using ultracentrifugation (UC) and size exclusion chromatography (SEC) did not change the overall structure. Some non-EV particulates were observed in the SEM sample, which was isolated using UC. The TEM sample which was isolated using SEC showed very little impurities. Using DLS as a complementary technique, the EBC-derived EVs were measured to be approximately 122 nm as is shown in Supplementary figure S-1. This data agrees with the size distribution measured manually of 90 EBC-derived EVs that were observed using Cryo-SEM which is also shown in Supplementary figure S-1. The broad size distribution reflects the heterogeneity in sizes of the EVs from the sample. Further characterisation of the breath condensate EVs was done using western blot where bands for CD63 and CD81 were probed (see Supplementary Figure S-7). WI38 cell-derived exosomes were used as a positive control to show the exosome specific marker (CD81) was present. This was compared to EVs isolated using the same method of isolation, which also showed CD63 specific biomarker.

Further confirmation of the binding to antibodies was shown using SPR which was used with an InP/ZnS QD-AB (where AB = Anti-CD63) conjugate. This was immobilised onto a secondary AB-functionalised gold surface. The QDs were used to show an amplified response in the binding of the antibody to the surface as opposed to using just the antibody which gave rise to lower responses. Supplementary figure S-2 (D) shows the high resolution transmission electron microscope (HR-TEM) image of the InP/ZnS QDs in toluene. The absorption and emission properties of the QDs used are shown in Supplementary figure S-2 (A) and (B) respectively. The original properties of the QDs were retained after ligand exchange, with minor shifts in the photoluminescence (PL) spectra upon ligand exchange and conjugation. Furthermore, the absorption peak at 590 nm seems to have disappeared completely post-conjugation to the AB. The confirmation of the conjugation of the AB to the InP/ZnS came from the increase in size as measured using DLS. This is shown in Supplementary Figure S-2 (C) where the original water-soluble and ligand-exchanged QDs have a hydrodynamic size of 37.8 nm. After conjugation, this size increases to 68.1 nm. There is also a change in the zeta potentials which indicates a change in the surface environment of the particles Supplementary Table S-3. Additionally, after conjugation and purification, the PL quantum yields (QY) of the QDs increased from 7.32 to 13.12%.

**Figure 2:**
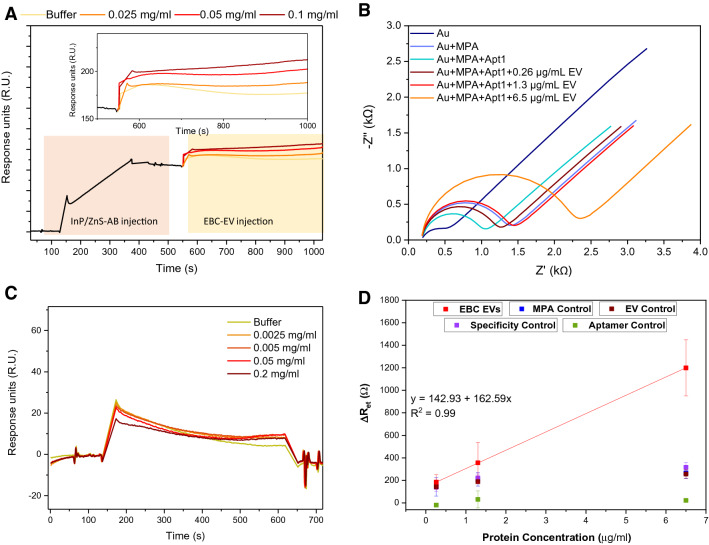
(**A**) SPR response from injection of EBC EVs onto a surface functionalised with InP/ZnS-AB; (**B**) Nyquist plots of impedance spectra obtained in a solution comprised of 10 mmol/L PBS and 150 mmol/L NaCl at pH 7.4 containing 5 mmol/L Fe(CN)_6_^3−^/ Fe(CN)_6_^4−^. (**C**) SPR response of the control surface functionalised with no InP/ZnS-AB. (**D**) EIS calibration curve showing the average change in charge transfer resistance ($\Delta \hbox {R}_{\mathrm{et}}$) for the log of each concentration of EV from the $\Delta \hbox {R}_{\mathrm{et}}$ value for Apt1 immobilised to the surface. Data also includes the control points from Supplementary table S-6 for better visualisation. Note that EBC EVs is exhaled breath condesate extracellular vesicles and MPA is mercaptoproprionic acid. Error bars are in accordance with the standard deviation of three separate measurements.

The SPR response to the capture of the conjugate gave rise to a response of roughly 200 R.U. as seen in Fig. 2A. After this, the EVs isolated from the breath condensate were flowed through the system at different protein concentrations. This gave rise to a subsequently higher response with higher concentrations of the EBC-derived EVs injected as more bind to the AB captured on the surface. A control was also run to test for the non-specificity of the secondary AB which showed that there was close to no binding of the EVs to the secondary AB on its own (Fig. 2C). Supplementary Figure S-4 also shows a correlation between the changing concentration of the EV sample and the change in R.U. for the respective samples. These values have been summarised in Table 1.

EIS was also carried out to detect the presence of EVs through binding of a complementary amino-modified CD63 aptamer (Apt1) that targets the CD63 exosomal membrane protein. The amino-modified Apt1 was immobilised via EDC/NHS chemistry to a mercaptopropionic acid (MPA) alkanethiol-self-assembled monolayer (SAM), directly bound onto the surface of the gold electrode. A Nyquist plot was taken at each step of immobilisation: the gold electrode (Au), Au+MPA, Au+MPA+Apt1 and finally Au+MPA+Apt1+EV, using three different concentrations of EV (Fig. 2B). The average charge transfer impedance values ($\hbox {R}_{\mathrm{et}}$) for both experimental and control measurements reported in this investigation was analysed and the experimental results are summarised in Table 2. The $\hbox {Fe(CN)}_{6}^{3-}$ $\hbox {Fe(CN)}_{6}^{4-}$ redox couple was chosen due it having a fast electron transfer rate. At high frequencies the kinetics of electron exchange become evident, hence the increase in $\hbox {R}_{\mathrm{et}}$ is caused by components bound to the gold surface inhibiting the electron transfer between electrolyte and electrode. At low frequencies, the electrode is driven by diffusion, hence the Warburg impedance increases represented as the slope of $45^{\circ }$ on the Nyquist plot. When MPA is bound to the gold surface, $\hbox {R}_{\mathrm{et}}$ increases by an average of 703.2 $\Omega$ from bare gold. The addition of this forms a SAM layer which prevents charge transfer between electrolyte and solution. Upon the addition of Apt1, $\hbox {R}_{\mathrm{et}}$ decreases significantly. This is caused by the negatively charged sequences aligning the MPA layer through repulsive interactions.

Three concentrations of EBC-derived EVs (0.26, 1.3, 6.5 μg/mL) were sequentially bound to the immobilised gold surface and $\hbox {R}_{\mathrm{et}}$ increased from lowest to highest concentration, caused by the increasing number of large protein molecules inhibiting charge transfer. A calibration curve was plotted to show the exponential correlation of increased $\hbox {R}_{\mathrm{et}}$ and concentration of EVs, shown in Fig. 2D. This confirmed that not only were the EVs captured with high specificity but that $\hbox {R}_{\mathrm{et}}$ is concentration-dependent. Control experiments were completed to validate these results, where 10 mmol/L PBS buffer (phosphate buffered saline) replaced each immobilisation layer, with their $\hbox {R}_{\mathrm{et}}$ values reported in Supplementary Table S-6. Firstly, Apt1 was replaced by 10 mmol/L PBS buffer (Supplementary figure S-5(a)). This showed no EV binding due to the absence of an aptamer, confirming the presence of the CD63 proteins on the EVs and that Apt1 has the correct sequence to target this. Next, PBS was put in place of EVs, shown in Supplementary figure S-5(b). This showed an insignificant response compared to when concentrations of EVs are present in the system, showing the successful capture of EVs.

**Table 1:** Averaged experimental values of the difference in response units ($\Delta$R.U.).

Protein concentration of EBC EVs (mg/mL)	$\Delta$R.U.
0.025	12.1709 ± 0.3534
0.05	26.4321 ± 0.7153
0.1	35.6479 ± 0.5181

**Table 2:** Averaged experimental values of charge transfer resistance ($\hbox {R}_{\mathrm{et}}$) and percentage errors for each component when added to the immobilisation layer.

Layer	$\hbox {R}_{\mathrm{et}}$, Z’ ($\Omega$)	Error / (%)
Au	438.90 ± 126.84	7.6728
Au+MPA	1142.10 ± 222.74	1.7364
Au+MPA+Apt1	841.76 ± 411.50	3.1860
Au+MPA+Apt1+0.26 μg/mL EV	1026.70 ± 405.51	2.0341
Au+MPA+Apt1+1.3 μg/mL EV	1198.40 ± 410.08	1.5134
Au+MPA+Apt1+6.5 μg/mL EV	2040.80 ± 502.28	0.4247

Further control experiments consisted of replacing MPA with 10 mmol/L PBS buffer. The results for this are shown in Supplementary figure S-5(c). Though there is no MPA, there is a small response in $\hbox {R}_{\mathrm{et}}$ from both Apt1 and also the different concentrations of EVs. However, this is non-specific binding to the now available and reactive gold surface sites. As this binding was independent to the excess concentrations of EV, it is therefore an inaccurate representation of EV capture with high specificity. Supplementary figure S-5(d) further proves the high specificity of Apt1 to the CD63 proteins on the membrane of the breath-derived EVs. Apt2 was chosen as it had the same length as Apt1 but with a different sequence. Although there was a small response in $\hbox {R}_{\mathrm{et}}$ between Apt2 and the different concentrations of EVs, this was not concentration-dependent and is trivial when compared to the response when using Apt1, especially when paralleling the highest concentration of EV added thereby emphasising the high specificity of Apt1 for the breath-derived EVs. These control results are further shown in Fig. 2D along with the calibration curve for three different protein concentrations of EVs. The significant increase in resistance values for the higher concentrations of EVs is clearly visible here when compared to each of the controls shown which have negligible changes.

## Conclusions

In conclusion, EBC-derived EVs and exosomes were isolated using size-exclusion columns and physically characterised using imaging techniques such as TEM, Cryo-SEM and DLS. Their morphology was spherical, and their sizes were between 50–170 nm. The ability of methods such as SPR and EIS towards detection of dilute concentrations of breath-derived EVs was also demonstrated by targeting the CD63 protein that is known to exist on the membrane of exosomes (sub-population of EVs). EIS was able to show a larger change between three different concentrations of the EVs which makes this label-free method the most suitable for the detection of dilute concentrations. Further potential of this work can extend to detection of clinically relevant populations of these EVs that are easily isolated from a non-invasive source for the detection of pulmonary diseases.

## Methods

### Breath sampling

The breath sampling was done as a proof-of-concept by the researcher. Subject provided written informed consent prior to participation.

### Chemicals and materials

All chemicals and materials were purchased from Sigma Aldrich unless specified otherwise.

### EVs isolation

Prior to collection of the condensate, the subject did not consume food or drink water for 2 h. The condensate was then collected by breathing out through the mouth every 3 s and in through the nose every 10 s. The breath was collected in a Eppendorf tube that was attached to a 1 mL syringe. The Eppendorf tube and 1 mL syringe were wrapped in a frozen ice gel pack. The breathing process was done for approximately 30 min ensuring the entire apparatus stayed frozen and then aliquoted into small vials for EVs isolation. The EBC was collected into an Eppendorf tube and diluted with 1 $\times$ phosphate buffered saline (up to 1 mL of PBS; Thermo Fisher, cat. 10010023). This was then filtered using a 0.22 μm syringe filter after which the EVs were isolated using qEV size exclusion chromatography (Izon Science Ltd.). Fractions 7, 8, 9 and 10 were mixed and concentrated using a 30 kDa centrifugal filter and re-diluted in 200 μL PBS. Protein concentration in the EBC-derived EVs sample was measured by using absorbance at 280 nm at NanoDrop^®^ND-1000 UV–Vis spectrophotometer (Thermo Fisher Scientific) and consisted of 0.023 mg/mL.

### EV isolation for Cryo-SEM

Initially, the sample was centrifuged for 10 min at 300$\times$*g* following next cycle of centrifugation at 3000$\times$*g* for 15 min to get rid of debris. Supernatant was collected for future analysis. In order to isolates EVs, the sample was run through the filter (0.22 μm) and then ultracentrifuged for 30 min at 10,000$\times$*g*. The supernatant was collected and placed into a clean 10 mL polycarbonate bottle with cap assembly, 10.4 mL, $16\times 76$ mm ( Beckman Coulter, cat. 355603) and then went under next cycle of centrifugation (Beckman Coulter Optima L-100 XP Ultracentrifuge) for another 90 min at 70,000$\times$*g* using type 70.1 Ti rotor, fixed Angle, titanium, $12 \times 13.5$ mL from Beckman Coulter Life Sciences (cat. 342184). After the last cycle, the pellets (EVs) were resuspended in 1 $\times$ PBS, pH 7.4 making a final volume of 110 μL. Protein concentration in the breath EVs sample was measured by using absorbance at 280 nm at NanoDrop^®^ND-1000 UV–Vis spectrophotometer (Thermo Fisher Scientific) and consisted of 1.34 mg/mL.

### Synthesis of AB conjugated InP/ZnS quantum dots

The method used here is according to a previously published procedure by Tessier et al.^15^ Indium(III)chloride (0.45 mmol) and zinc(II)chloride (2.2 mmol) were mixed with 5.0 mL of oleylamine. This mixture was heated to $120\,^{\circ }\hbox {C}$ under vacuum for 2 h. The atmosphere was switched to nitrogen and the temperature was set to $180\,^{\circ } \hbox {C}$ at which point, the tris-(diethylamino)phosphine (1.6 mmol) was added rapidly. The InP core growth was allowed to proceed for 20 min, after which sulfur (2.2 mol/L) in tri-octylphosphine (1 mL) was added to the cores for shelling over 10 min. At 60 min, the temperature was ramped to $200\,^{\circ } \hbox {C}$. At 120 min, Zn(undecylenate)2 (1 g) in 4 mL of octadecene was slowly injected dropwise over a period of 10 min after which the temperature was increased to $220\,^{\circ } \hbox {C}$. At 150 min, 0.7 mL of TOP-S was added over a period of 10 min and the temperature was further ramped to $240\,^{\circ } \hbox {C}$. At 180 min, Zn(undecylenate)2 (0.5 g) in 2 mL of octadecene was added slowly and the temperature was ramped to $260 \,^{\circ } \hbox {C}$. At 210 min, the reaction was terminated with rapid cooling to room temperature and subsequent dilution in toluene. The QDs were then washed using ethanol for precipitation and re-dispersed in toluene. This method yielded red-emitting QDs. The method used below for ligand exchange was based on a previous procedure published by Dobhal et al.^12^ 0.30 g of Mercaptosuccinic acid (MSA) was stirred in 1 mL of toluene for 15 min after which 1 mL of 10 mg/mL QDs were added. 1 mL of ammonium hydroxide (30%) and 1 mL of Milli-Q water were added after 1 min. This was left to stir for 2 h. The coloured aqueous layer was purified by precipitating in ethanol and centrifuging. The clear supernatant was discarded and the pellet was re-dispersed in 1 mL of Milli-Q water. The water-soluble QDs were stored in the dark at $4 \,^{\circ } \hbox {C}$. For the conjugation of the QDs to an antibody, the following AB was used: CD63 Monoclonal AB (Ts63) from Thermo Fisher Scientific, catalog 10628D, RRID AB_2532983. 50 μL of 0.05 M of *N*-hydroxysuccinimide (NHS) and 0.02 M of 1-ethyl-3-(3-dimethylaminopropyl)carbodiimide (EDC) was mixed with 1 mL of 1 mg/mL QD-MSA for 10 min. Then, 10 μL of a 0.5 mg/mL AB-CD63 was added. This was left to stir for 2 h. The conjugate (InP/ZnS-AB) was washed using a 30 kDa Amicon centrifugal filter twice with water and re-dispersed in 1 mL of water.

### Transmission electron microscopy

TEM2100F (JEOL Ltd.) was used for imaging purposes. EV samples were drop-casted on the formvar side of the grid which had been previously plasma cleaned. After 15 min, the sample was stained for 6 min using 4.5% uranyl acetate. After this, the grid was washed twice with Milli-Q water and left to dry for 30–45 min before loading into the microscope.

### Cryo-scanning electron microscopy

For cryo-SEM imaging we used a JSM6500F (JEOL Ltd.), equipped with a 97 μA emission gun and a ALTO 2500 (GATAN UK) cold stage maintained below $-\,12\,^{\circ } \hbox {C}$. Specimens were imaged at low acceleration voltages of 10 kV, and working distances of 8.4 mm. Specimens were cryo-fixed using a manual drop plunging where a 10 μL drop of a sample of EVs in PBS were set on top of a sample stage, maintaining its droplet shape. This was manually plunged into liquid nitrogen. The frozen droplet was transferred into a freeze fracture system. A cooled knife was used to fracture the droplet exposing the inner part of the drop. The sample was coated with 5–10 nm of platinum and transferred into the SEM.

### UV–Vis spectroscopy

The Cary50 Bio (Agilent technologies) was used for absorbance measurements.

### Photoluminescence spectroscopy

The FLS980 (Edinburgh Instruments) was used for all PL measurements. All the InP QDs samples were excited at 480 nm. Prior to making a QY measurement, it was ensured that the QD sample absorbance at the excitation wavelength of 480 nm was below 0.1 absorbance units to avoid any self-absorbance effects. All PLQY calculations were done using the integrating sphere and a direct excitation method.

### Surface plasmon resonance

This protocol is based on a modified protocol used by Dobhal et al.^12^ SPR measurements were done using the Biacore X100 (GE Healthcare, Life sciences). 0.1 M HEPES, 1.5 M NaCl, 30 mM EDTA, 0.5% v/v Surfactant P20 (HBS-EP, GE Healthcare, Life sciences) buffer was the running buffer and the flow rate used was 5 μL/min. The secondary antibody used was Goat anti-Mouse IgG1 Cross-Adsorbed Secondary AB from Thermo Fisher Scientific, catalog A10538, RRID AB_2534038. This was immobilised onto a CM3 chip at a concentration of 26 μg/mL using 0.4 M 1-ethyl-3-(3-dimethylaminopropylcarbodiimide) (EDC) and 0.1 M *N*-hydroxysulfosuccinimide (NHS) in water at a response of 2000 units (R.U). The injection of the QD-antibody conjugates (at the same concentration) gave rise to a response of 200 R.U. The EVs (at varying protein concentrations) were then flowed for 18 min. The response from the blank solution with no EVs and just antibody was subtracted to obtain responses from the change in EV concentration. To account for refractive index changes from the different buffer systems, one of the flow cells on the chips was used as a reference. This experiment was run three times with each concentration flowed through the same CM3 chip which had the secondary antibody immobilised to it.

### Electrochemical impedance spectroscopy

This protocol is based on a modified method used by Queirós et al.^16^ EIS experiments were carried out using a three-electrode cell: working electrode is gold (Au), counter electrode is platinum (Pt), reference electrode is silver/silver chloride (Ag/AgCl). Measurements were carried out using a PalmSens3^®^potentiostat with a 5 mmol/L $\hbox {Fe(CN)}_{6}^{3-}$/$\hbox {Fe(CN)}_{6}^{4-}$ electrolyte solution in PBS electrolyte buffer solution (10 mmol/L PBS with 150 mmol/L NaCl electrolyte solution at pH 7.4). Experiments were carried out at room temperature pressure and all solutions were purged under nitrogen gas for 15 min prior to measurements. Impedance spectra was collected between a frequency range of 0.1–10000.0 Hz range. Pre-cleaning of each electrode was monitored using cyclic voltammetry (CV). Each electrode was pre-treated in 5 mL of a degassed solution 0.1 mol/L NaOH for 200 scans ( 8 min) before being rinsed with Milli-Q water and dried under nitrogen. The electrode was then polished using 0.3 μmol/L alumina for 2 min before being rinsed with Milli-Q water and dried under nitrogen. This process was repeated using 0.05 μmol/L alumina and further rinsed with Milli-Q water and ethanol. The electrode was sonicated in ethanol followed by sonication in Milli-Q water for 5 min each. The Au electrode was cleaned with 3 mL of 0.5 mmol/L $\hbox {H}_{2}\hbox {SO}_{4}$ for 60 scans. After cleaning, the electrode was immobilised in MPA for 1 h and was washed with ethanol and Milli-Q water and dried with nitrogen. The Au-electrode was then immersed in an aqueous solution of 0.2 mol/L NHS and 0.05 mol/L EDC for 1 h before being washed with Milli-Q water and dried with nitrogen. The aptamer used for EIS was synthesised by Integrated DNA Technologies, named amino-modified CD63 aptamer (Apt1): 5-NH2-(CH2)6-CACCCCACCTCGCTCCCGTGACACTAATGCTA-3. The Au-electrode was then immersed overnight in a 5 μmol/L aptamer solution before being washed with Milli-Q^®^water. The electrode was finally immobilised in 50 μL of different concentrations of breath-derived EVs in 10 mmol/L PBS solutions, starting from the lowest concentration, for 30 min each. Each experiment was run independently three times. Analysis of the Nyquist plots obtained in each measurement was carried out using the EIS Spectrum Analyser programme, using the Randles equivalent electric al circuit with an unweighted function and the Nelder-Mead (NM Simp) algorithm. This gave both impedance values and relative estimated errors of the calculated parameters.

## Supplementary information

Supplementary Information.
