# Supplementary material for: Isolation, characterisation and detection of breath-derived extracellular vesicles
Source: Sci Rep. 2020 Oct 15;10:17381. doi: 10.1038/s41598-020-73243-5 (PMC7566616; doi:10.1038/s41598-020-73243-5)
Supplement: Supplementary file 1 — Supplementary Information. [file 41598_2020_73243_MOESM1_ESM.docx]

The supplementary material on the publication entitled “Isolation, characterisation and detection of breath-derived extracellular vesicles”

*Garima Dobhal ^1^, Amrita Datta ^2^, Deanna Ayupova ^2^, Paul Teesdale-Spittle ,^3,4^ and Renee V. Goreham ^1*^*

1. School of Mathematical and Physical Sciences, University of Newcastle, Callaghan 2308, Australia
2. School of Chemical and Physical Sciences, Victoria University of Wellington, Wellington 6012, New Zealand
3. School of Biological Sciences, Victoria University of Wellington, Wellington 6012, New Zealand
4. Centre of Biodiscovery, Victoria University of Wellington, Wellington 6012, New Zealand

***** Correspondence: renee.goreham@newcastle.edu.au; Tel.: + 61 4 49138252

**Supplementary figure S-1**: (Left) Size distribution of 90 EBC derived EVs as measured using Cryo-SEM. (Right) Size by intensity measured using DLS.


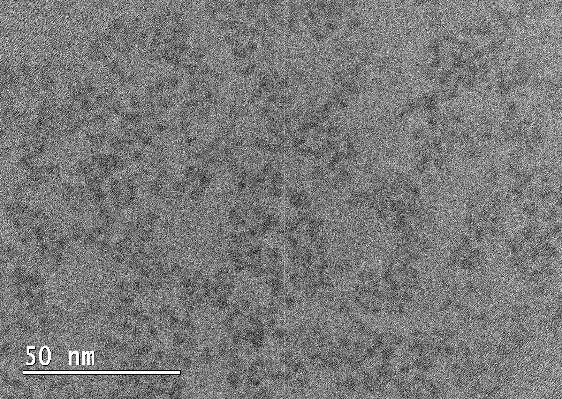


A

B

C

D

**Supplementary figure S-2.** (A) Absorbance profiles of the QDs used. (B) Emission profiles when excited at 480 nm. (C) Change in hydrodynamic size of the QDs post-conjugation as measured using DLS. (D) HR-TEM image of InP/ZnS QDs in toluene.

**Supplementary table S-3:**

Zeta potential and PLQY values for the InP/ZnS QDs used for SPR

| Sample | Zeta Potential (mV) | PLQY (%) |
| --- | --- | --- |
| InP/ZnS | - | 25.45 |
| InP/ZnS-MSA | -40.1 | 7.32 |
| InP/ZnS-AB | -14.7 | 13.12 |


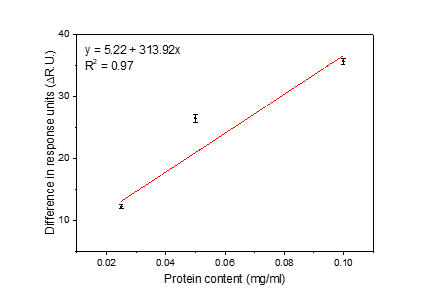


**Supplementary figure S-4**: Calibration curve for SPR of EBC EVs following a linear fit.


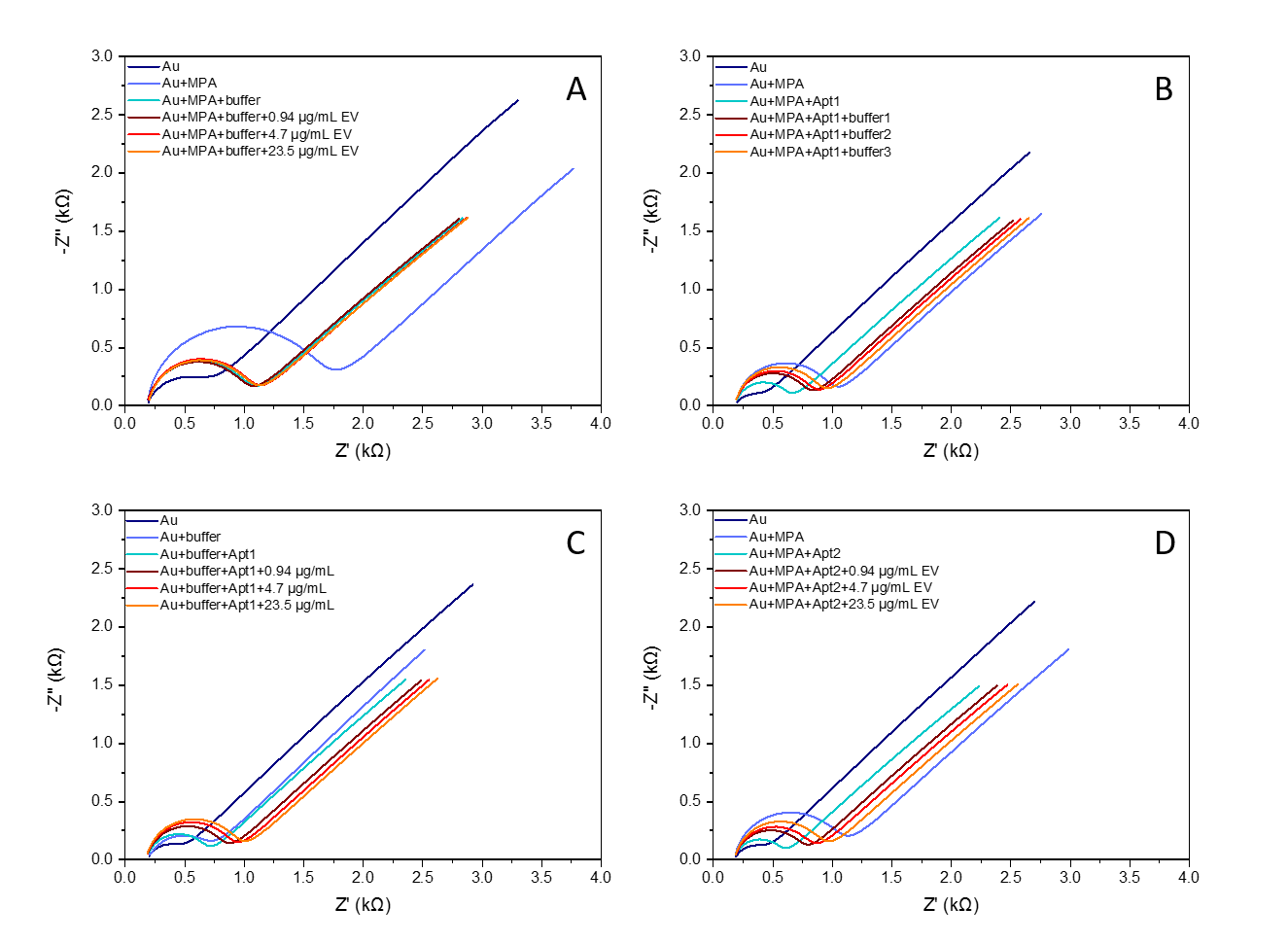


a

b

c

d

**Supplementary figure S-5.** Nyquist plots of impedance spectra representing the control experiments for when 10 mM PBS buffer replaces (a) Apt1; (b) three different concentrations of EVs; (c) MPA. (d) shows Apt1 being replaced by Apt2.

**Supplementary table S-6:**

Average charge transfer impedance values (R_et_) and percentage errors associated with supplementary figure S-1 for the control experiments.

| Aptamer control | | | |
| --- | --- | --- | --- |
| *Layer* | *R_et_, Z’ (Ω)* | | *Error/ %* |
| Au | 603.64 | | 8.6035 |
| Au+MPA | 1480.10 | | 1.6382 |
| Au+MPA+buffer | 884.35 | | 2.7010 |
| Au+MPA+buffer+0.26 µg/mL EV | 865.51 | | 2.7610 |
| Au+MPA+buffer +1.3 µg/mL EV | 916.23 | | 2.5793 |
| Au+MPA+buffer +6.5 µg/mL EV | 906.52 | | 2.9655 |
| Concentrations of EV control | | | |
| *Layer* | *R_et_, Z’ (Ω)* | *Error/ %* | |
| Au | 309.05 | 2.9931 | |
| Au+MPA | 825.45 | 1.7987 | |
| Au+MPA+Apt1 | 487.69 | 3.7671 | |
| Au+MPA+Apt1+buffer1 | 633.91 | 2.5297 | |
| Au+MPA+Apt1+buffer2 | 676.91 | 2.5360 | |
| Au+MPA+Apt1+buffer3 | 744.07 | 2.2623 | |
| MPA control | | | |
| *Layer* | *R_et_, Z’ (Ω)* | *Error/ %* | |
| Au | 469.12 | 5.2463 | |
| Au+buffer | 561.53 | 2.4700 | |
| Au+buffer+Apt1 | 530.82 | 2.8088 | |
| Au+buffer+Apt1+0.26 µg/mL EV | 673.69 | 2.2189 | |
| Au+buffer+Apt1+1.3 µg/mL EV | 741.62 | 1.9306 | |
| Au+buffer+Apt1+6.5 µg/mL EV | 797.70 | 1.8634 | |
| Specificity control | | | |
| *Layer* | *R_et_, Z’ (Ω)* | *Error/ %* | |
| Au | 269.95 | 5.2904 | |
| Au+MPA | 899.66 | 2.1855 | |
| Au+MPA+Apt2 | 445.61 | 4.2470 | |
| Au+MPA+Apt2+0.26 µg/mL EV | 599.20 | 2.8066 | |
| Au+MPA+Apt2+1.3 µg/mL EV | 667.49 | 2.6989 | |
| Au+MPA+Apt2+6.5 µg/mL EV | 761.71 | 2.2459 | |

**Supplementary methods:**

**Cell-derived exosome isolation**

WI 38 cells were grown to 75% confluency under standard conditions, washed in PBS and incubated overnight in serum-free media. After 24 hours, vesicle-containing pellets were obtained from conditioned culture medium by ultracentrifugation as described by Lobb *et al*.[1] The pellets were resuspended in cold filtered (0.22 µm filter, Merck Millipore) PBS, pH 7.4, concentrated by centrifugal concentration (Amicon Ultra-4, Merck Millipore) and exosomes isolated by SEC Stranska *et al*.[2]

**Exosomes purification and characterisation by Western Blot.**

Samples from breath and conditioned cell culture media were assayed using western blotting to confirm the isolation procedure produced purified exosomes. Isolation of exosomes was performed as previously described, with blotting undertaken on samples from pooled fractions 7, 8, and 9 of SEC isolation using qEV original columns and from ultracentrifugation. Negative control samples free of exosomes were also assayed.

Samples were suspended in lysis buffer (50mM Tris-HCl pH 8.0, 150mM NaCl, 1% NP-40) and subjected to two rounds of vortex vigorously (10 s) and cooling on ice (20-30 min). Samples were finally centrifuged at 15,000 x*g* for 10 min. The pellet was discarded and the protein concentration of the supernatant was determined using a micro-BCA assay (Pierce BCA Protein Assay Kit) according to the manufacturer’s instructions. Samples containing approximately 20 µg of protein were loaded on a 10% acrylamide gel, and proteins separated by electrophoresis.

The western blot was performed according to the protocol described by Lim *et al.* [3] with minor amendments: 5% bovine serum albumin was used as a blocking agent; the membrane was probed with anti-CD63 at 1:250 dilution (TS63; Invitrogen, ref. 10628D), anti-CD81 at 1:250 dilution (M38; Invitrogen, ref.10630D) and secondary mouse monoclonal secondary antibody conjugated to horseradish peroxidase at 1:5000 dilution (Santa Cruz, sc-2005). The membrane was visualized on an Amersham Imager 600 (General Electric).


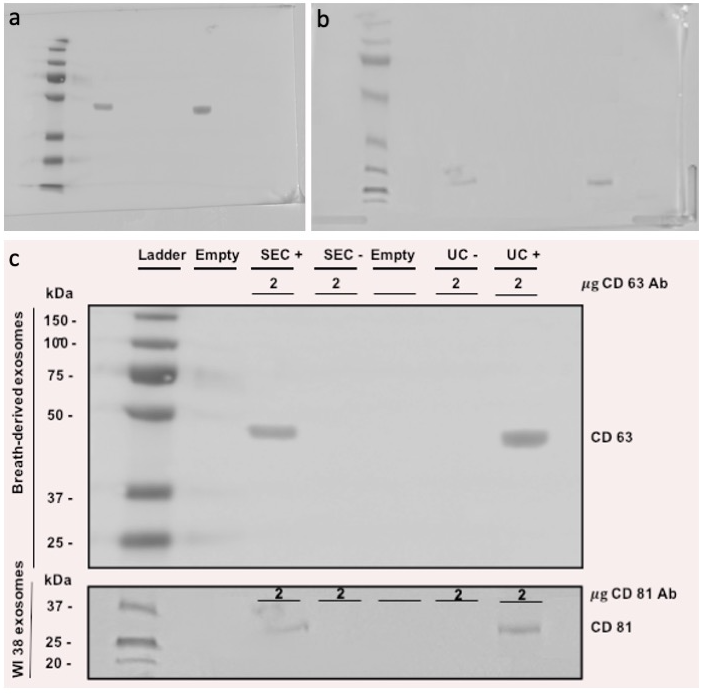


**Supplementary figure S-7.** Western blot analysis using common exosome markers CD63 and CD81 in exosomes derived from breath and WI38 cells. Samples that contain exosomes from size exclusion chromatography or ultracentrifugation are labelled SEC+ and UC+, respectively. (a) shows the raw gel image for CD63 marker and (b) shows the raw gel image for CD81 marker. (c) Exosome-free negative controls are also shown. These are samples of matching total protein content to the exosome samples but are from exosome-free size exclusion or ultracentrifugation fractions, and are labelled SEC- and UC-. The markers demonstrate that the methodology isolates exosomes from breath equivalently to the established isolation from cell culture conditioned media. Total loaded protein lysate ~ 20 µg/lane for all samples including negative controls. Blotting was performed using 2 μg/mL of anti-CD63 and anti-CD81. The goat anti-mouse secondary antibody was used at a concentration of 0.08 μg/mL.

## References

1. Lobb RJ, Becker M, Wen SW, et al (2015) Optimized exosome isolation protocol for cell culture supernatant and human plasma. J Extracell Vesicles 4:27031. https://doi.org/10.3402/jev.v4.27031

2. Stranska R, Gysbrechts L, Wouters J, et al (2018) Comparison of membrane affinity-based method with size-exclusion chromatography for isolation of exosome-like vesicles from human plasma. J Transl Med 16:1. https://doi.org/10.1186/s12967-017-1374-6

3. Lim J, Choi M, Lee H, et al (2019) Direct isolation and characterization of circulating exosomes from biological samples using magnetic nanowires. J Nanobiotechnology 17:1. https://doi.org/10.1186/s12951-018-0433-3
